# Proteomic Analysis of the Secretome of *Cellulomonas fimi* ATCC 484 and *Cellulomonas flavigena* ATCC 482

**DOI:** 10.1371/journal.pone.0151186

**Published:** 2016-03-07

**Authors:** Warren W. Wakarchuk, Denis Brochu, Simon Foote, Anna Robotham, Hirak Saxena, Tamara Erak, John Kelly

**Affiliations:** 1 Department of Chemistry and Biology, Ryerson University, Toronto, Ontario, Canada; 2 Human Health Therapeutics Program, National Research Council Canada, Ottawa, Ontario, Canada; Korea University, REPUBLIC OF KOREA

## Abstract

The bacteria in the genus Cellulomonas are known for their ability to degrade plant cell wall biomass. *Cellulomonas fimi* ATCC 484 and *C*. *flavigena* ATCC 482 have been the subject of much research into secreted cellulases and hemicellulases. Recently the genome sequences of both *C*. *fimi* ATCC 484 and *C*. *flavigena* ATCC 482 were published, and a genome comparison has revealed their full spectrum of possible carbohydrate-active enzymes (CAZymes). Using mass spectrometry, we have compared the proteins secreted by *C*. *fimi* and *C*. *flavigena* during growth on the soluble cellulose substrate, carboxymethylcellulose (CMC), as well as a soluble xylan fraction. Many known *C*. *fimi* CAZymes were detected, which validated our analysis, as were a number of new CAZymes and other proteins that, though identified in the genome, have not previously been observed in the secretome of either organism. Our data also shows that many of these are co-expressed on growth of either CMC or xylan. This analysis provides a new perspective on Cellulomonas enzymes and provides many new CAZyme targets for characterization.

## Introduction

Strains of the Cellulomonas genus were shown almost 40 years ago to produce extracellular enzymes that could efficiently degrade pretreated sugarcane bagasse [1]. As interest in enzymatic saccharification grew for production of fuel ethanol from biomass, there was a corresponding interest in microbial cellulases, and those from Cellulomonas were an obvious choice to study. The enzymes were shown to be both free in the supernatant and cellulose bound [2]. The composition of the cellulases produced was shown to be a complex mixture [3, 4], including some proteins which were clearly proteolytic breakdown products [5]. The advent of molecular cloning soon revealed that indeed there were many genes for cellulases and xylanases in the type strain *C*. *fimi* ATCC 484 [6–12]. These genes were identified using expression cloning from shotgun libraries which was a remarkably successful approach for Cellulomonas and for many other bacterial cellulase systems. However, given the limitations of expression cloning at the time, it was not evident that all of the potential Cellulomonas secreted plant cell wall degrading enzymes had been identified.

With advances in proteomic technology it became possible to probe secretomes directly for protein content. An elegant approach using gel-free activity based profiling was applied to the *C*. *fimi* secretome and permitted the identification of a new β-1,4-glycanase [13]. A more traditional approach was applied to identify proteins in the secretome *C*. *flavigena*., A number of potential cellulase and xylanase proteins were identified by this method, but because at that time the genome had not been sequenced, the corresponding genes could not be easily identified [14].

Dramatic changes to the cost and availability of microbial genome sequencing has changed our understanding of the CAZyme content of bacteria. Increased sequencing data has led to better bioinformatics prediction of CAZymes, and the CAZy database [15] has grown exponentially as a result. Various actinobacteria related to Cellulomonas have been sequenced and their CAZymes are now included in the CAZy database. For example, 43 glycoside hydrolase (GH) enzymes are predicted in *Thermobifida fusca* and various *Streptomyces sp*. have around 159 GH enzymes. The genome of *C*. *fimi* ATCC484 has 109 GH enzymes and that of *C*. *flavigena* ATCC482 has 89 [16]. In order to make sense of these multi-enzyme systems for biomass degradation, it is important to understand under what conditions and with what kind of induction and regulation are different subsets of CAZymes expressed. The secretome of both *T*. *fusca* and a *Streptomyces species* have been examined by modern proteomics techniques [17, 18], but to date, no similar analysis of the secretome of Cellulomonas species has been completed.

Presented here are the results of a mass spectrometry study to identify the proteins secreted by *C*. *fimi* and *C*. *flavigena* during growth on carboxymethylcellulose (CMC) as well as a soluble xylan fraction. Many known *C*. *fimi* CAZymes were detected as were a number of other CAZymes and proteins that, though identified in the genome, have not previously been observed in the secretome of either organism. Furthermore, we show here that expression of many of these proteins appears to be dependent on the growth medium (CMC or xylan).

## Material and Methods

### Growth of bacteria and preparation of proteins

*C*. *fimi* ATCC484 and *C*. *flavigena* ATCC482 were grown at 30°C, with shaking at 150 rpm in low salt Luria broth (0.5 g NaCl, 5 g Yeast extract, 10 g of bacto-tryptone per litre) supplemented with either 0.2% medium viscosity carboxymethylcellulose, or 0.2% soluble birch wood xylan (extracted from birch wood xylan with 60°C water, then freeze dried) or 0.5% glucose. CMC and xylan were autoclaved in the culture media, glucose was added from a sterile stock solution in water. Plate grown cells were used to inoculate 25 ml starter cultures in low salt Luria broth with 0.2% glucose as carbohydrate. Growth was carried out in 1L baffled shake flasks with 200 ml of media in each flask. The A600 nm was monitored and cultures were processed when the A600 reached ~ 3 (30 hours of growth). Culture supernatants were concentrated 20 fold by ultrafiltration on 10,000 mwco polyethersulfone membranes (VWR) Cellulase and xylanase activity was measured with a 5 mg/mL suspension of AZCL-hydroxyethylcellulose and AZCL-xylan (Megazyme Corp. Ireland) in 50 mM sodium phosphate, 150 mM NaCl, pH 6 buffer at 37°C various times. Release of soluble fragments was measured in clarified reaction supernatants at A540 nm as suggested by the manufacturer. Biological replicates were grown exactly the same way, but from a different starter culture on a different day.

The supernatant (100 mL) was first filtered through glass fibre and then freeze-dried. The proteins were then dissolved in 10 ml of water and ice-cold TCA was added to 16.7% final concentration to precipitate the proteins. After mixing, the TCA precipitation mixture was kept on ice for 15 minutes, and then the proteins were collected by centrifugation at 10,000 x g for 20 min at 4°C. The pellets were washed twice with ice cold 10% TCA, twice with acetone, and then briefly dried in a fume hood. The pellets were solubilized in 60 mM Tris-HCl buffer pH 7.0. The samples were diluted to 62.5 mM Tris-HCl pH 6.8, 2% SDS, 5% 2-mercaptoethanol and 10% glycerol, using a 3X loading buffer, prior to loading on gels.

### Analysis of proteins by mass spectrometry

The supernatant proteins were resolved on a 12% SDS-PAGE mini gel. Protein bands were visualized using Biosafe Coomassie Blue stain. For analysis, each of the sample containing lanes were cut into 25 equal bands using a gel cutter from about 150kDa to just above the dark smear at the bottom of the gel. Selected protein gel bands were excised and destained by gentle shaking in 100 mM ammonium bicarbonate (ABC), 30% acetonitrile (ACN). This step was repeated 2–3 times until the gel band was fully destained. The gel band was dehydrated in ACN, reswollen in 10 mM dithiotreitol in 50 mM ABC and incubated for 1 hour at 54°C. Excess DTT solution and replaced with 55 mM iodoacetamide in 50 mM ABC, enough to cover the band. The samples were left in the dark at room temperature for 1 hour after which the excess iodoacetamide solution was removed. The gel bands were dried and reconstituted in 20 ng/μL trypsin in 100 mM ABC sufficient to just cover the gel pieces. The vials were sealed and incubated overnight at 37°C.

The in-gel digests were analyzed by nanoLC-MS/MS using a QTOF Ultima hybrid quadrupole time-of-flight mass spectrometer coupled to a NanoAquity UPLC system (Waters, Milford, MA). The digests were desalted on a 180 μm x 20 mm 5-μm Symmetry® C18 trap (Waters) and separated on a 100 μm x 100 mm, 1.7 μm BEH130 C18 column (Waters) using the following gradient conditions: hold at 1% mobile phase B (0.2% formic acid in acetonitrile) for 1 minute, increase to 45% B in 18 minutes, increase to 85% B in 2 minutes and then decrease to 1% B in 1 minute. The flow rate was 400 nL/min and the eluate was directed to the nanoelectrospray interface of the Q-TOF. Incoming ions (m/z 400–1600) were surveyed at a rate of 1 scan per second and MS/MS spectra were acquired on doubly, triply and quadruply charged ions (up to 3 precursor ions selected per survey scan). The digests of the second biological repeats were also analyzed by nanoLC-MS/MS on a LTQ-Orbitrap-XL hybrid mass spectrometer (ThermoFisher Scientific). The digests were separated using similar chromatographic conditions to those described above. A survey scan (m/z 400–2000, 60k resolution) was performed in the FTMS analyzer and data-dependent turbo MS/MS scans on the top 3 multiply charged ions were acquired in the linear ion trap (dynamic exclusion of 180 seconds).

The resulting MS/MS spectra were submitted to the Mascot® search engine (Matrix Science Ltd, London, U.K.) and searched against the *C*. *fimi* ATCC484 or *C*. *flavigena* ATCC482 complete genome as well as NCBI nr database (May 11^th^, 2013 version). The searching parameters were as follows: enzyme = trypsin, missed cleavages = 1, variable mods = oxidation and carbamidomethyl, peptide tolerance = 1.5Da and MS/MS tolerance = 1.2Da. A custom Java application was used to analyze the Mascot search results and to generate a presence/absence table for all identified proteins within the *C*. *fimi* and *C*. *flavigena* proteome. Only peptides which meet or exceed the threshold values for 95% confidence level leading to Mascot non-probabilistic protein scores (sum of peptide scores) greater than 50 were considered in the analysis.

## Results and Discussion

Using genome data, the number of GH enzymes in different species can be predicted. We compared the genomes of four actinobacteria (*C*. *fimi (CFi)*, *C*. *flavigena CFl)*, *S*. *coelicolor* (SC) and *T*. *fusca* (TF)) and found a similar number of members of different GH subtypes in each species (Table 1). A notable exception was 16 predicted GH-10 xylanases in Cfl compared to 5 or less in the other actinobacteria. We then undertook a study to identify GH enzymes at the protein level in the secretome of *CFi* and *CFl* grown on common laboratory polysaccharide substrates, carboxymethylcellulose, and soluble oat-spelt xylan. Soluble polysaccharides were chosen for this study as insoluble substrates are known to tightly bind some secreted enzymes through interactions with CBMs. Proteins were collected and analyzed from two biological replicates.

**Table 1:** Known cellulase and xylanase families in related actinobacteria. Families are from the CAZy database (http://www.cazy.org/Glycoside-Hydrolases.html). The total GH number does not include pectin lyases.

Organism	CBM33 (AA10)	GH1	GH3	GH5	GH6	GH9	GH10	GH11	GH16	GH48	GH74	Total GH
***C*.*fimi***	1	1	11	3	4	4	5	1	3	1	1	109
***C*. *flavigena***	4	1	3	3	3	5	16	3	0	1	0	89
***S*. *coelicolor***	2	5	9	5	3	2	2	1	5	1	1	159
***T*. *fusca***	2	4	2	3	2	2	2	1	0	1	1	43

**Fig 1:**
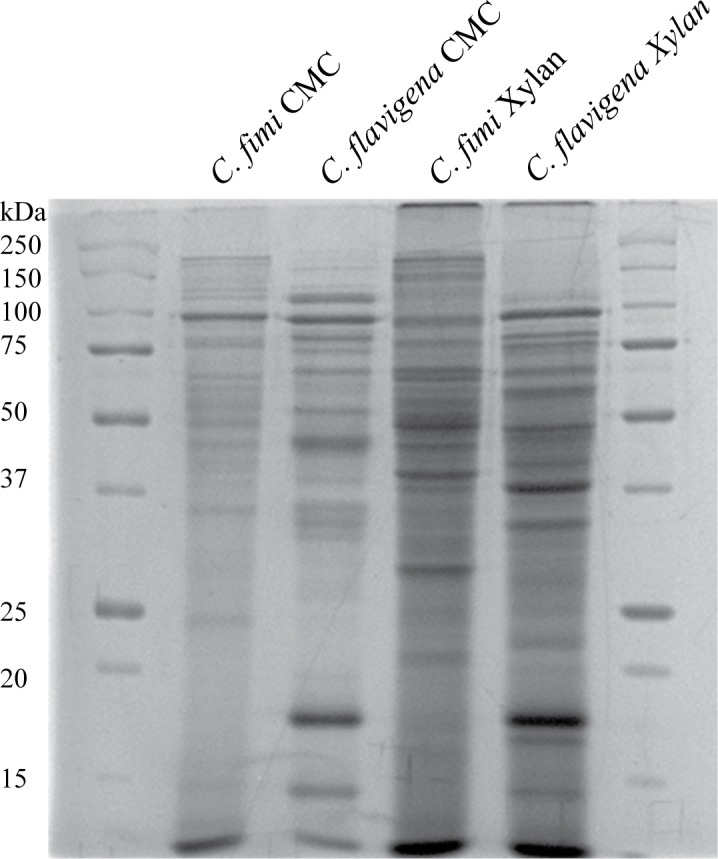
Example of the SDS-PAGE that was used for GelLC analysis of supernatant proteins. Lanes 1 and 6: BioRad All-blue SDS-PAGE standards; Lane 2: proteins recovered from the supernatant of CMC grown *C*. *fimi* ATCC484 cells; Lane 3; proteins recovered from the supernatant of CMC grown *C*. *flavigena* ATCC482 cells; Lane 4: proteins recovered from the supernatant of soluble xylan grown *C*. *fimi* ATCC484 cells; Lane 5: proteins recovered from the supernatant of soluble xylan grown *C*. *flavigena* ATCC482 cells.

We grew the bacteria in low salt Luria broth supplemented with either 0.2% CMC, or 0.2% birch wood xylan or 0.5% glucose. There was no detectable cellulase or xylanase activity in the glucose grown cultures, whereas both cellulase and xylanase activity were measured in both of the induced cultures (data not shown). This is consistent with the published activity profiles of these strains (3, 14). The proteins were concentrated using a trichloroacetic acid precipitation, and then analysed on polyacrylamide gels. Fig 1 shows a typical one-dimensional gel that was used for the proteomic analysis. For analysis, each of the sample containing lanes were cut into 25 equal bands using a gel cutter from about 150kDa to just above the dark smear at the bottom of the gel. We analysed tryptic fragments liberated from the gel slices by LC-MS.

**Fig 2:**
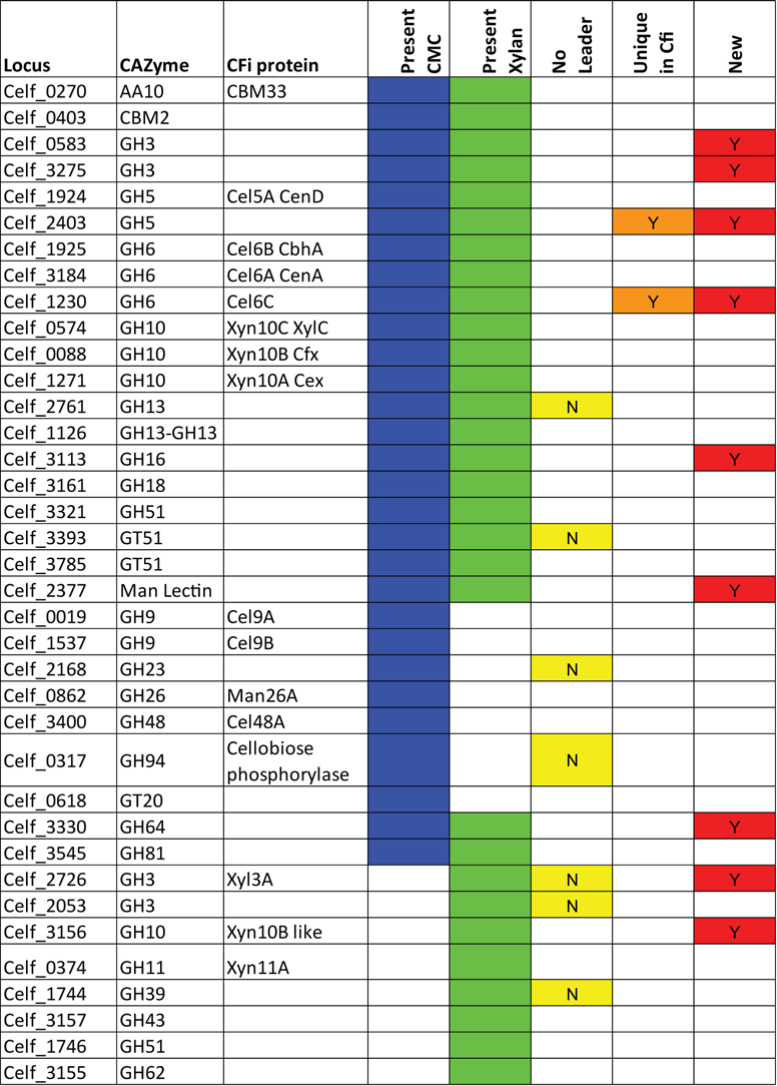
CAZyme content from *C*. *fimi* culture supernatants. The proteins are identified by CAZy family, and if they have been previously characterized, the designation is given in CFi protein column. The proteins expressed in media with CMC are indicated with a blue box or those from media with xylan with a green box. The yellow box identifies proteins that have no bioinformatically identified secretion leader (SEC or TAT). The tan box indicates this protein is unique to *C*. *fimi*, and the red box identifies a new cellulase or xylanase. The data are arranged by enzyme family for each group: both substrates, CMC only, followed by Xylan only.

**Fig 3:**
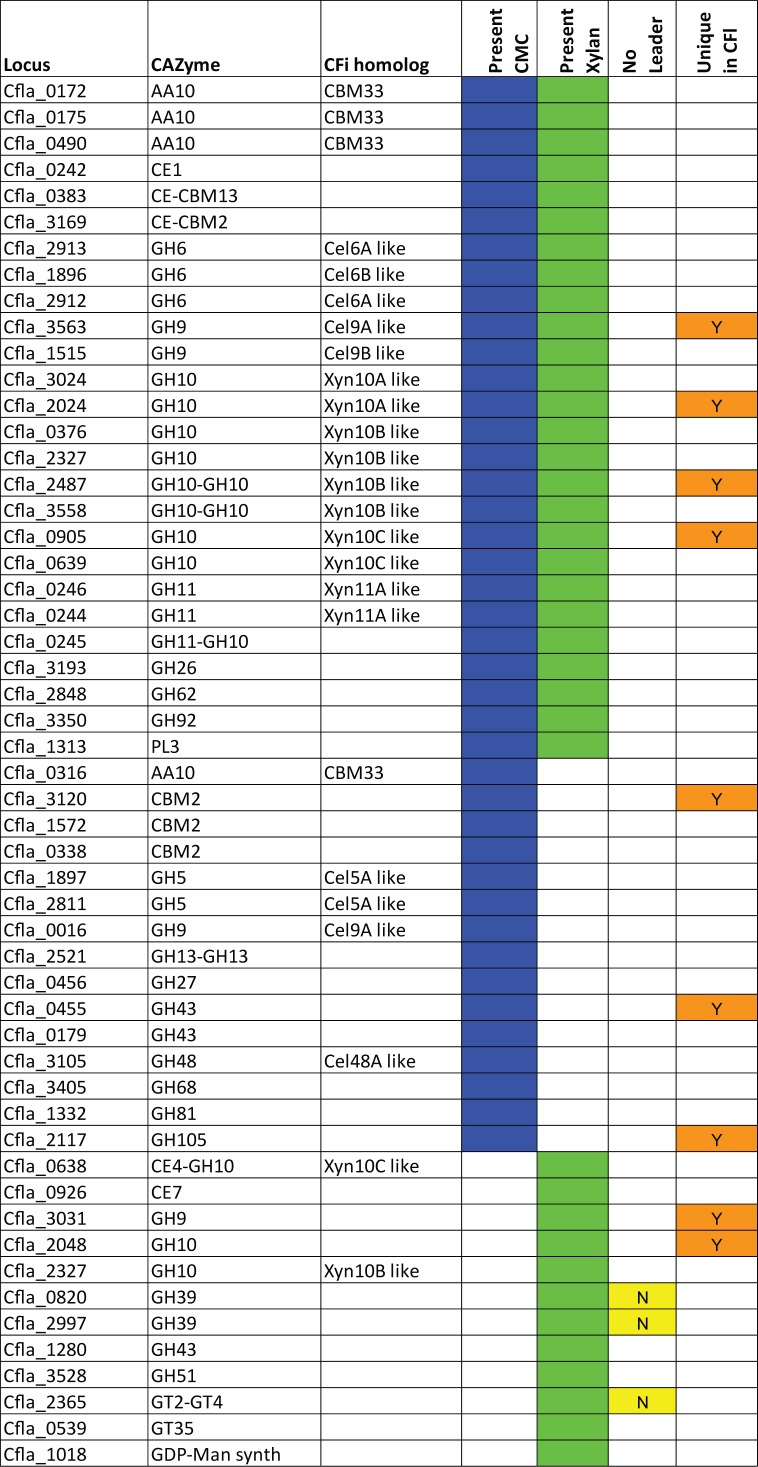
CAZyme content from *C*. *flavigena* culture supernatants. The proteins are identified by CAZy family, and if they have been previously characterized, the designation is given in CFi homologue column. The proteins expressed in media with CMC are indicated with a blue box or those from media with xylan with a green box. The yellow box identifies proteins that have no bioinformatically identified secretion leader (SEC or TAT). Since so few proteins have been characterized from *C*. *flavigena* supernatants we have not indicated the “new” proteins as we did for *C*. *fimi*. The tan box indicates this protein is unique to *C*. *flavigena*. The data are arranged by enzyme family for each group: both substrates, CMC only, followed by Xylan only.

Protein identification was performed as indicated in the methods and the resulting data are summarized in S1 Table. In order to be counted, a protein had to be found in at least 1 of the 2 supernatants (CMC or Xylan induced) in both biological replicates. A total of over 600 proteins were identified from this analysis many of which were known cellular components like ribosomal proteins, or enzymes from central metabolism. These cellular proteins arose from cell lysis in the culture during the growth and were simply concentrated along with all the other proteins in the supernatant. The proteins which were considered as being extracellular were divided by their genome annotations and those that were considered intracellular were ignored in the analysis. The CAZyme i.d. results obtained for *C*. *fimi* and *C*. *flavigena* on the two growth media are summarised in Figs 2 and 3, respectively. We have also indicated when we could not bio-informatically identify a secretion leader of the SEC or TAT type for a protein seen in the preparations.

### CAZymes secreted by *C*. *fimi*

#### Cellulases

The CAZyme content of the *C*.*fimi* and *C*. *flavigena* supernatants is shown in Figs 2 and 3, which is organized by proteins found in both growth conditions, followed by CMC supernatant only, followed by xylan supernatant only. In CFi there were many known CAZymes being expressed. The cellulases: Cel6A (CenA)/Cel6B (CbhA), Cel5A(CenD), Cel9B (CenC), Cel48A(CbhB or CenE) were better represented in the CMC supernatant than in the xylan supernatant. Absent from the supernatants were the cellulase Cel9A (CenB) and the new GH9 members Cel9C/D. A new member of the Cel6 family, Celf_1230 (Cel6C) was easily detected in both conditions. This enzyme is unique to CFi and blast searches reveal no close homologues in other actinobacteria. We have cloned and characterized this enzyme, it is an endoglucanase and the biochemical characterization of this enzyme Cel6C will be published elsewhere (B. Hsu et al in preparation). We did not confidently identify the fourth predicted GH6 family member, except as single peptide, so it is not shown in the Figure. There is a second GH5 family member, Celf_2403 produced in both conditions. We have also cloned and expressed this protein but, so far at least, it has not demonstrated any enzymic activity (H.Saxena unpublished).

#### Additional Glucanses

Proteins which may be β-glucanases and have not yet been described from *C*. *fimi* include a GH3 which based on sequence comparisons [19] is likely a cellodextrinase (Celf_0583), there is also a member of the AA10 (formerly CBM33) family of the copper-dependent lytic polysaccharide monooxygenases. There were also proteins from GH16, GH18, and GH64 which are potential β-glucanases that have not been characterized from *C*. *fimi*. In addition we also detected in the CMC supernatant a GH81 β-1,3-glucanase, and a GH94 cellobiose phosphorylase, which lacked typical SEC or TAT leader sequences, but which could be involved in glucan degradation.

#### Xylanases

As expected the xylanases were more represented in xylan supernatant than CMC, but Xyn10A (aka Cex) was seen in similar amounts in both conditions, possibly reflecting its mixed activity on cellobiosides as well as xylan [20]. Xyn10B (XylC) and Xyn10C (Cfx) were predominantly in the xylan supernatant, while a new Xyn10 (Celf_3156) was seen exclusively in xylan supernatants. The GH11 Xyn11A is also exclusively seen in the xylan supernatant. Enzymes of a known support function for xylan degradation were also detected, these include α-L-arabinofuranosidases from GH43 (only 1 of 7 predicted), GH51 (3 of the 4 predicted) and one from GH62. The GH51 enzyme, Celf_3249, was seen in both supernatants but at very low levels. There were also 3 GH3 family members detected, one of which, Celf_2726, is a recently characterized β-xylosidase [21] which would be involved in further breakdown of the xylan hydrolysis products.

### CAZymes secreted by *C*. *flavigena*

#### Cellulases

The cazome from CFl was more complex with 52 CAZymes (Fig 3) compared to only 38 CAZymes detected from CFi (Fig 2). This is interesting as the genome of CFi has 20 more CAZymes than CFl but shows a lower complexity under our induction conditions. Far fewer CAZymes have been characterized from CFl compared to CFi, so the number of characterized enzymes detected in our supernatants is low. As there are so few characterized enzymes from the genome strain of CFl we chose to label them as “like” CFi enzymes based on sequence homology where possible. There have been no GH6 enzymes described from CFl, but based on sequence comparisons Cel6A/B are related to the CFi equivalents. The characterized GH5 enzyme from *C*. *flavigena* CDBB 531 [22] appears to be in the genome strain (Cfla_1897 which is Cel5A like or known as CelB). The characterized CBP105 endoglucanase from *C*. *flavigena* CDBB531 [23], is related to Cfla_0016 and Celf_0019 (Cel9A), and is indicated on the figure. A detailed comparison of the genome encoded CAZyme content of these two strains has been published [16] and will not be repeated here.

CFl has 4 predicted members of the AA10 (formerly CBM33) family of the copper-dependent lytic polysaccharide monooxygenases, 3 of which we observed, 2 of which appear to be produced only when grown on CMC, and one which is also produced during growth on xylan. This a major difference from CFi where there is only one AA10 family member which is observed in both conditions. Presumably these are an important component of the degradative machinery for Cellulomonas as has been seen in other microorganisms [24].

#### Xylanases

It is notable that in the CFl cazome are a large number of GH10 xylanases, some of which are tandem catalytic domains, in fact 12 GH10 catalytic domains were observed out of a possible 16 predicted in the genome. In terms of xylan degradation the accessory enzymes from GH39 (β-xylosidase) and GH/43/51 (α-L-arabinofuranosidases) are all expressed and a total of 6 of these were expressed in contrast to only 3 α-L-arabinofuranosidases in CFi. There were also 5 carbohydrate esterases or CE domains observed, which contrasts the CFi samples where a single CE7 and 3 CE-domains were seen. It appears that CFl has indeed a potent xylan degrading enzyme repertoire, certainly much more complex than CFi.

#### Additional CAZymes of interest

There are a number of enzymes observed which are not thought of as being part of the cellulolytic or xylanolytic enzyme complex. They can be seen in Table 2. There is a pectate lyase from PL3, a GH27 family member of unknown function only in the CMC sample, a GH68 levansucrase, a GH32 (active onfructose containing glycans), a GH92 α-mannosidase, and a GH105 unsaturated rhamnogalacturonyl hydrolase. In addition a number of peptidoglycan active enzymes are also seen such as GT51, the peptidoglycan transglycosylating enzymes, as well as a GH23 lytic transglycosylase, and peptidoglycan binding proteins.

**Table 2:** Detected cellulase and xylanase enzymes compared to the prediction.

Organism	CBM33 (AA10)	GH3	GH5	GH6	GH9	GH10	GH11	GH16	GH48	GH74
*C*.*fimi*	1/1	4/11	2/3	3*/4	2/4	4/5	1/1	1/3	1/1	1
*C*. *flavigena*	4/4	0/3	2/3	3/3	4/5	12/16	3/3	0	1/1	0

* The fourth GH6 Celf_0233 was present only as a single peptide and so is not counted in the total.

### Observed differences in CAZymes between the strains

**Fig 4:**
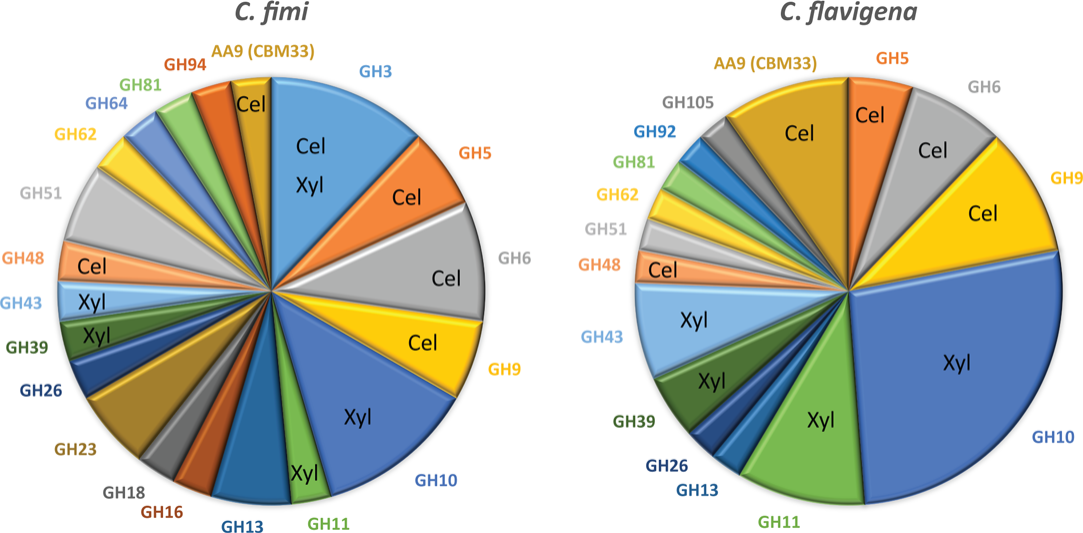
Summary of the observed CAZyme content. In order to facilitate visual comparison of the supernatant CAZymes, we summarized the content by enzyme family in a pie chart. Each family that had at least one member is plotted as a different coloured section of the chart. Those families that have known activities toward cellulose or xylan degradation are labelled with “Cel” or “Xyl”. The sections are proportional to the number of family members that were identified in each supernatant.

Based on our data presented in Figs 2–4, we saw 38 GHs in CFi and 52 in CFl. The tally of observed GHs in known cellulase/xylanase families shows that 20/35 in the genome are found for CFi, and 28/39 for CFl (Table 2). One major difference in that total comes from the 12 GH10 proteins observed in CFl compared to only 4 GH10 seen in CFi. Another is the presence of 4 CFl expressed CBM33 (AA10) copper dependant lytic mono-oxygenases, compared to just one in CFi. In CFi supernatants 10/38 CAZymes are from families that are involved in other carbohydrate linkage degradation/formation. What was common to both strains were the presence of the accessory enzymes for acetyl-xylan breakdown, GH39/43/51, and in CFl carbohydrate esterase modules. This suggests that the control of expression of many CAZymes is not tightly regulated by carbon source, and that perhaps expression is leaky since pure carbohydrate sources in nature would be rare. It remains to be determined if control of expression of these enzymes is mediated by transcription factors as has been seen in the Clostridia [25].

#### Other CAZymes of interest

In CFl there were 8/52 CAZymes involved in other carbohydrate linkage specificity. One example in both CFi and CFl was a tandem GH13 catalytic module, normally associated with α-glucan degradation. A GH26 β-mannanase was also present in both strains, along with a GH92 α-mannosidase, and GDP-mannose synthase seen only in CFl. The expression of these may simply be constitutive and not linked to “induction” by the carbon sources we used for this study. Other examples only in CFi were enzymes which act on peptidoglycan, GT51, GT20, and GH23. It is not surprising to see peptidoglycan remodelling enzymes, as they would be present in any growing cell, and under conditions where increased export was needed they could be up-regulated to help with increased export machinery being incorporated in membrane.

#### A brief description of non-CAZYME proteins observed in our analysis

Our analysis of both CFi and CFl supernatants identified a large number of other proteins which fit our MASCOT cut-off score of 50. These are shown in S1 and S2 Figs. We have classified these as proteases, periplasmic/extracellular, hypothetical and other. S2 Table lists a summary of the total number of those proteins which met our analysis criteria. Proteases were known to give rise to heterogeneity of cellulases in CFi [3], but these were never isolated or examined for their precise role in CAZyme production or action on biomass. Since the specificity of these proteases is not known the relevance to CAZyme function remains unknown.

The periplasmic/extracellular group of proteins represent a diverse group of proteins. Common to both species were extracellular solute binding proteins (ESBP) of families 1,3 and 5, which are typically membrane anchored lipoproteins associated with different classes of nutrient uptake[26]. *It could be that some of these are involved in oligosaccharide uptake from the digested carbon source*. Members of ESBP family 1 include carbohydrate ABC transporter associated substrate binding (CFi 7 members of which 2 are expressed only on xylan, CFl also 7 members, 1 of which is expressed only on xylan). Based on available literature (26), the members of ESBP family 3 do not appear to be involved in carbohydrate assimilation, but could be domains involved in peptidoglycan remodelling. There is one protein from CFl, Cfla_3696 which is annotated as a peptidoglycan binding protein, which again could be involved in cell wall remodelling. *These binding proteins in Gram-positive bacteria would be membrane anchored lipoproteins that presumably facilitate nutrient uptake*.

The group of proteins listed as “other” shows some proteins in common which are associated with carbohydrate utilization. Xylose and L-arabinose isomerase, and xylulokinase all involved in pentose metabolism. In addition there was a large secreted protein annotated simply as FN3 domain repeat protein, which was found in both strains, expressed in both carbon sources. These 2 proteins share 61% identity over more than 2000 amino acids, and blast search revealed it to be highly conserved in 11 Cellulomonas database entries, as well as many other actinobacteria. The large FN3 domain containing protein may be of further interest as it appears to be a highly conserved actinobacterial protein which is secreted by both of these strains. Celf_2339/Cfla_2198 are 2036 aa in length with 5 repeats of an FN3 domain. Many secreted hydrolases contain FN3 domains, and in *Clostridium thermocellum*, tandem FN3 domains from a Cbh9A (Cthe_0413) have been shown to disrupt the surface of cellulose fibres [27]. Again it is tempting to speculate that a protein that large could provide a scaffold for other secreted proteins, or could indeed interact with carbohydrate substrates through the FN3 domains.

## Conclusions

Despite the availability of the genome sequences of many biomass degrading bacteria, we are only beginning to understand how the protein composition of the secretome changes in response to a given feedstock. For this study, we chose to use soluble lab substrates as feedstocks in order to get a more complete snapshot of the secretome without the need to process residual substrate to recover bound enzymes. In the work presented here the accounting for the predicted CAZymes certainly shows significant CAZyme expression from both strains including most of the predicted cellulase and xylanase including all of the predicted AA10 enzymes which are most beneficial for utilization of insoluble substrates.

Generally speaking, the Gel-LC-MS approach worked well and identified many (but not all) of the expected proteins. It should be pointed out that some of the expected CAZymes have been identified by molecular cloning approaches only and have not been characterized in actual supernatants from the bacteria. The overall protein composition was reproducible between biological replicates. Furthermore, the analysis of the second biological repeat digests performed on the Q-Tof Ultima and the LTQ-Orbitrap XL mass spectrometers identified the same major proteins though the overall peptide coverage and scores were better for the latter instrument (S1 Table). This is attributable to its faster acquisition speed. Not many intracellular proteins were observed and some of those that were detected are known to be involved in biomass utilization, and so may have been truly up-regulated under our growth conditions. Overall, we believe this data describes the basic secretome for these important biomass degrading bacteria on two relevant carbohydrate sources.

As a follow-up to this study into the secretomes of *C*. *fimi* and *C*. *flavigina*, we are working to determine the specificity of some of the newly identified CAZymes (Saxena et al in preparation). We are also now examining the glycoproteome of Cellulomonas. Many cell surface proteins as well as secreted proteins appear to be mannosylated as has been seen in other actinobacteria [28–30]. Our basic secretome data will provide an excellent point of reference for this comparative glycoproteome analysis between CFi and CFl and related actinobacteria.

## Supporting Information

S1 Fig Non-CAZyme content from *C*. *fimi* culture supernatants. The proteins are identified by genome annotation (if there was one). The proteins expressed in media with CMC are indicated with a blue box or those from media with xylan with a green box. The yellow box identifies proteins that have no bioinformatically identified secretion leader (SEC or TAT). The tan box indicates this protein is unique to *C*. *fimi*, and if there was a homologue in CFl this is indicated with the locus ID in the last column.
(TIF)

S2 Fig Non-CAZyme content from *C*. *flavigena* culture supernatants. The proteins are identified by genome annotation (if there was one). The proteins expressed in media with CMC are indicated with a blue box or those from media with xylan with a green box. The yellow box identifies proteins that have no bioinformatically identified secretion leader (SEC or TAT). The tan box indicates this protein is unique to *C*. *flavigena*, and if there was a homologue in CFi this is indicated with the locus ID in the last column.
(TIF)

S1 Table Proteomic data collected from *C*. *fimi* and *C*. *flavigena*. Protein identification was performed as indicated in the methods and the resulting data are summarized here. *C*. *fimi* and *C*. *flavigena* protein homologues are indicated along with annotation from NCBI database searches. In order to be counted, a protein had to be found in at least 1 of the 2 supernatants (CMC or Xylan induced) in both biological replicates. The data are arranged by decreasing MASCOT score and all of the peptide counting information is also given. The colour indicators are green if a supernatant sample contained peptides from that protein, or red if the sample did not have peptides from that sample.
(XLS)

S2 Table Protein information used to generate the figures. All of the data used to generate protein information figures is in the spreadsheet. The sheets contain data for the individual figures shown in the paper and in the supplemental material.
(XLSX)
