# Supplementary figures and images for: Proteomic Analysis of the Secretome of Cellulomonas fimi ATCC 484 and Cellulomonas flavigena ATCC 482
Source: PLoS One. 2016 Mar 7;11(3):e0151186. doi: 10.1371/journal.pone.0151186 (PMC4780727; doi:10.1371/journal.pone.0151186)

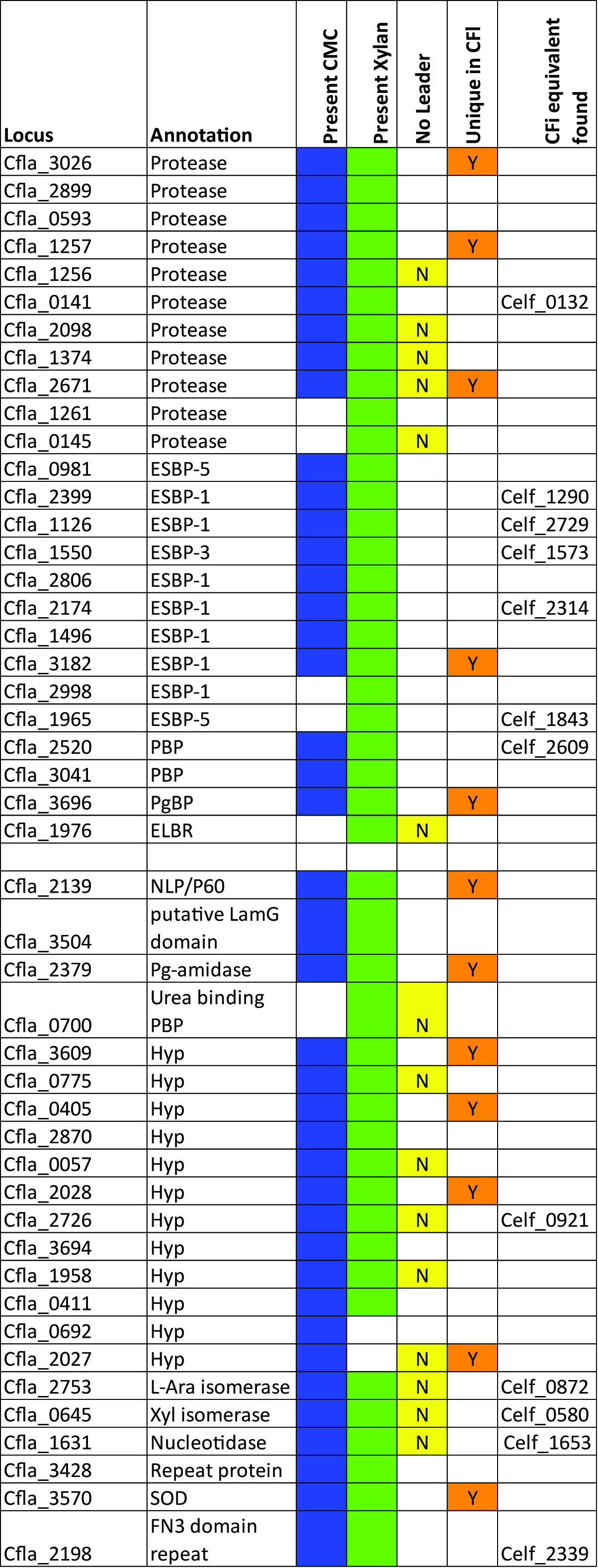

Supplement: S1 Fig — The proteins are identified by genome annotation (if there was one). The proteins expressed in media with CMC are indicated with a blue box or those from media with xylan with a green box. The yellow box identifies proteins that have no bioinformatically identified secretion leader (SEC or TAT). The tan box indicates this protein is unique to C. fimi, and if there was a homologue in CFl this is indicated with the locus ID in the last column. (TIF) [file pone.0151186.s001.tif]

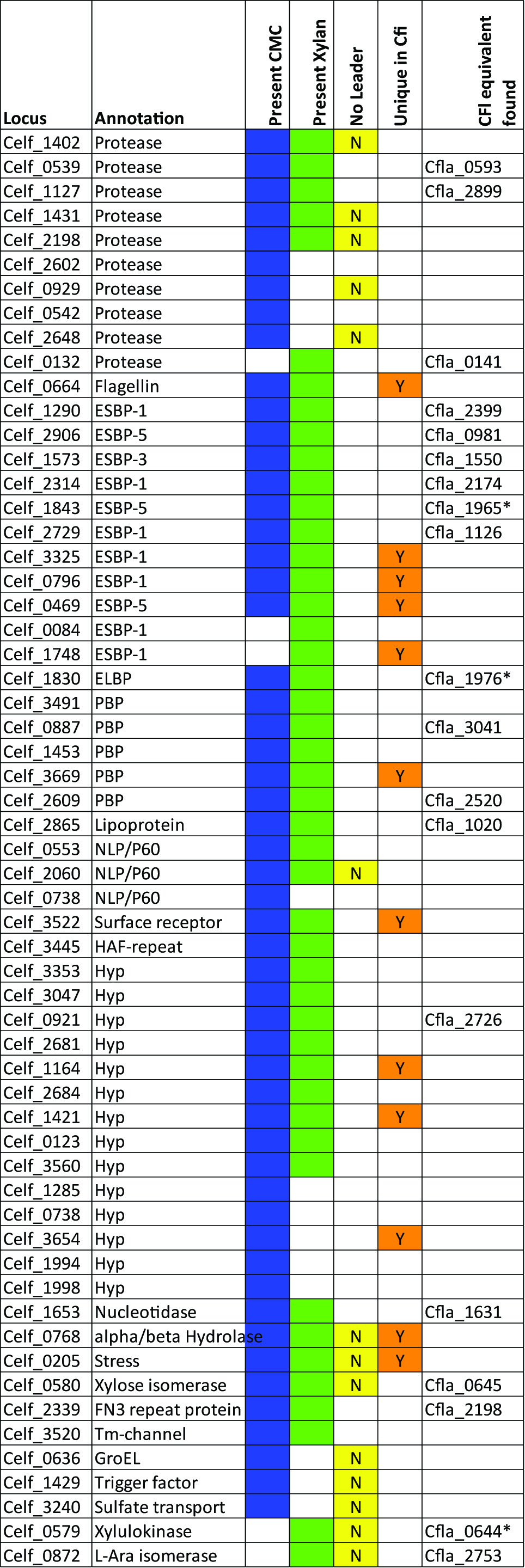

Supplement: S2 Fig — The proteins are identified by genome annotation (if there was one). The proteins expressed in media with CMC are indicated with a blue box or those from media with xylan with a green box. The yellow box identifies proteins that have no bioinformatically identified secretion leader (SEC or TAT). The tan box indicates this protein is unique to C. flavigena, and if there was a homologue in CFi this is indicated with the locus ID in the last column. (TIF) [file pone.0151186.s002.tif]
